# Usefulness and safety of preoperative portal vein embolization in older adults: A STROBE-compliant observational study

**DOI:** 10.1097/MD.0000000000048457

**Published:** 2026-04-24

**Authors:** Yuzuru Sakamoto, Shingo Shimada, Toshiya Kamiyama, Yoh Asahi, Akihisa Nagatsu, Tatsuya Orimo, Tatsuhiko Kakisaka, Hirofumi Kamachi, Daisuke Abo, Akinobu Taketomi

**Affiliations:** a Department of Gastroenterological Surgery I, Hokkaido University Graduate School of Medicine, Kita-ku, Sapporo, Japan; b Department of Diagnostic Imaging, Hokkaido University Graduate School of Medicine, Kita-ku, Sapporo, Japan.

**Keywords:** hepatectomy, older adults, portal vein embolization

## Abstract

With the continuing global trend of population aging, the number of older patients who require hepatectomy for malignant or benign liver diseases is steadily increasing. Postoperative liver failure remains one of the major causes of mortality after major hepatic resection, particularly among patients with limited hepatic reserve. Preoperative portal vein embolization (PVE) has been established as a safe and effective method to increase the future remnant liver volume (RLV), thereby improving postoperative outcomes. However, the regenerative capacity of the aged liver and the clinical benefits of PVE in older patients have not been fully clarified. This study aimed to assess the safety and efficacy of PVE in older patients compared with younger counterparts. We analyzed 113 patients who underwent PVE at Hokkaido University Hospital between 2000 and 2020. Patients were classified into 2 groups: 30 older patients (≥75 years; older group) and 83 younger patients (<75 years; younger group). We compared the patients’ characteristics, PVE-related factors, period from PVE to surgery, increase in RLV after PVE, and perioperative complications. Right hepatectomy was the most common surgical procedure (76.1%). The increase in RLV 2 weeks after PVE was comparable between the older (131.9%) and younger (130.3%) groups, and the period from PVE to surgery was also comparable (29 vs 26 days. respectively). Additionally, planned surgeries were completed successfully in all older patients. PVE-related complications were comparable, with recanalization of the embolic portal branch in 2 cases (6.7%) in the older group and 11 cases (13.3%) in the younger group. The liver failure rate, as a postoperative complication, was similar in the groups. Despite concerns regarding the diminished hepatic regenerative potential associated with aging, preoperative PVE effectively induces hypertrophy of the future remnant liver and may enable safe major hepatectomy in older patients. Although our findings suggest comparable outcomes between older and younger patients, these results should be interpreted with caution. PVE may be a safe and useful strategy in appropriately selected older patients requiring major liver resection.

## Graphical abstract

**Figure:**
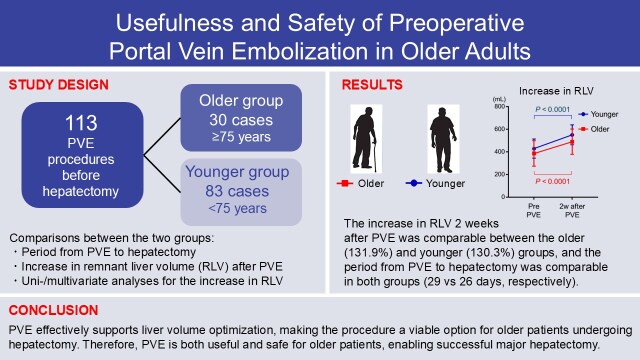

## 1. Introduction

A country’s demographic structure changes dramatically with increasing general population aging and declining birth rates.^[1]^ With the increase in the number of older people worldwide, the number of hepatectomies for this group is increasing.^[2,3]^ Hepatectomy is an important treatment option for long-term survival in patients with primary or secondary liver malignancies. However, the postoperative liver failure rate ranges from 8% to 32% and is still the major cause of death following major hepatectomy.^[4]^ Portal vein embolization (PVE) is considered when extended hepatic resection is required but the future liver remnant (FLR) volume is too small.^[5,6]^ The recommended FLR volume ranges from 20% to 40%, depending on the background liver status, and a small FLR volume is associated with poor surgical outcomes.^[7,8]^ PVE promotes hypertrophy in the non-embolized liver and increases the FLR function and volume.^[9]^ However, some patients exhibit insufficient hypertrophy of the non-embolized liver, with between 2.8% and 4.5% of patients unable to undergo surgery after PVE because of insufficient hypertrophy.^[10,11]^ A previous report also suggested that aging may suppress hepatic functional reserve in the FLR after PVE in patients with perihilar cholangiocarcinoma (PHCC).^[12]^ Moreover, as with all percutaneous transhepatic puncture procedures, various complications, such as pneumothorax, subcapsular hematoma, arterial puncture, pseudoaneurysm, hemobilia, and portal vein thrombosis, might occur with PVE.^[13]^ PVE is useful for safe major hepatectomy for malignant liver tumors and is widely performed. However, its usefulness and safety in older patients have not been investigated in detail, including changes in liver volume. In this study, we aimed to evaluate the usefulness and safety of preoperative PVE in older patients.

## 2. Materials and methods

### 2.1. Patients

Between January 2000 and December 2020, 113 patients underwent PVE for hepatectomy at the Gastroenterological Surgery I unit of Hokkaido University Hospital in Sapporo, Japan. The entire liver volume, liver resection volume, and tumor volume were calculated from contrast-enhanced computed tomography (CT) data using a 3-dimensional workstation (Virtual Place Lexus, Medical Imaging Laboratory, AZE, Tokyo, Japan or Synapse Vincent, Fujifilm Medical Co., Ltd., Tokyo, Japan). PVE was performed in patients with an effective liver resection rate (ELRR) > 60% using the following formula: [(liver resection volume − tumor volume)/(whole liver volume − tumor volume)] × 100.^[14]^ We performed bile duct drainage (generally, nasobiliary drainage) before PVE in patients with serum total bilirubin (T-bil) ≥ 2.0 mg/dL because a high serum T-bil level at the time of PVE is associated with poor hepatic hypertrophy.^[15]^ Accordingly, PVE was performed only for patients with T-bil < 2.0 mg/dL. This study was performed with the approval of the Institutional Board of Hokkaido University Hospital (No. 019-0458; approval date: March 24, 2020) and in accordance with the Helsinki Declaration guidelines. Informed consent was obtained as an opt-out format on the hospital website.

### 2.2. PVE (Percutaneous transhepatic portal vein embolization, PTPE)

The PTPE procedure has been described previously.^[16]^ Briefly, most patients underwent an ipsilateral approach; however, the contralateral approach was chosen when interventional radiologists deemed the ipsilateral approach unsuitable. Under local anesthesia, the intrahepatic portal vein was punctured under ultrasound (US)-guidance with an 18-gauge puncture needle (Create Medic Co., Yokohama, Japan). A 5.5-French sheath introducer (Introducer Set; Medikit Co., Tokyo, Japan) was inserted into the portal vein with a guidewire. Direct portography was performed to evaluate the vascular anatomy and measure portal venous pressure directly. Next, selective portography was performed with a balloon occlusion catheter (Selecon MP Catheter II; Terumo Co., Tokyo, Japan). The embolic material was absolute ethanol. Embolization was repeated until hepatic parenchymal enhancement disappeared, after which direct portography was performed to confirm the completion of PTPE. Portal venous pressure was again measured at the non-embolized lobe. Finally, the 5.5-French sheath was extracted by packing the puncture tract with a gelatin sponge torpedo (Spongel; Astellas Pharma Co., Tokyo, Japan).

### 2.3. Diagnoses and definitions

The diagnoses of primary tumors, disease progression, and resectability status were assessed on the basis of a patient’s general status, physical findings, serological test results, and imaging studies, namely contrast-enhanced CT, magnetic resonance imaging, and US. Surgical procedures were determined on the basis of liver function and general status, including disease extent.^[14]^ Anatomical resections, such as right or left bisegmentectomy and trisectionectomy were performed. Liver function was assessed on the basis of blood liver function test results, estimation of the indocyanine green retention rate at 15 minutes, and calculation of the technetium-99m-galactosyl human serum albumin (^99m^Tc-GSA) scintigraphy index. To evaluate the usefulness of preoperative PVE in older patients, the primary endpoint of the present study was the increase in the remnant liver volume (RLV) 2 weeks after PVE. The secondary endpoint was complications related to PVE and hepatectomy. Postoperative liver failure was recorded and graded in accordance with the International Society for Study of Liver Surgery grade.^[17]^

### 2.4. Comparison between the younger and older patients

The patients were divided into 2 groups on the basis of age. The older group comprised patients aged ≥ 75 years, and the younger group comprised patients aged < 75 years. We compared the patients’ characteristics, primary diseases, surgical procedures, embolized portal vein branches, period from PVE to surgery, increase in RLV measured by contrast-enhanced CT 2 weeks after PVE, and complications.

### 2.5. Portal venous flow volume (PV flow) before and after PVE

PV flow was measured 3 times per day using pulsed wave Doppler US (Toshiba SSA-700A (Aplio50), Toshiba Medical Systems Co., Ltd., Tokyo, Japan and LOGIQ P6 BT11, GE Healthcare Japan Co., Ltd., Tokyo, Japan) before PVE and on days 1, 3, 5, and 7 after PVE. PV flow was calculated according to the following formula: PV flow = portal velocity (measured by pulsed wave Doppler US) × π*r (r* = radius of the portal vein)2. The average of the 3 daily measurements was used as the representative PV flow value. The measurement site of the PV flow varied according to the planned resection: the umbilical portion for right hepatectomy, the anterior branch for left hepatectomy, and the posterior branch for left trisectionectomy.^[18]^

### 2.6. Statistical analysis

Categorical data were compared using the chi-square test. Continuous data were presented as median (range) or mean (± standard deviation) and compared between the younger and older groups using the Mann–Whitney *U*-test. Correlations were assessed using Spearman’s rank correlation coefficient. Univariate and multivariate analyses were performed using a logistic regression model. The optimal cutoff value for the platelet count was defined as the lowest value for (1 − sensitivity)2 + (1 − specificity)2, which corresponds to values with sensitivity and specificity closest to the (0, 1) point on the receiver operating characteristic curve. The optimal cutoff values for factors other than the platelet count were defined as the normal values in our hospital. *P* < .05 was considered statistically significant. All statistical analyses were performed with JMP 16 software (SAS Institute Inc., Cary) or GraphPad Prism 7 (GraphPad Software, Inc., La Jolla).

## 3. Results

### 3.1. Patient characteristics

The older group comprised 30 patients, and the younger group comprised 83 patients. The median age in the older group was 79 (75–84) years and that of the younger group was 64 (40–74) years. No significant differences were found between the groups for sex, laboratory data (i.e., platelet count, prothrombin time, serum albumin, cholinesterase), and liver function tests, such as indocyanine green retention rate at 15 minutes or ^99^mTc-GSA scintigraphy index (Table 1).

**Table 1:** Patients’ characteristics in the older and younger groups.

		Older	Younger	*P*-value
		n = 30	n = 83	
Age (yr)		79 (75–84)	64 (40–74)	**<.0001**
Sex				
	Male (%)	20 (66.7)	60 (72.3)	.564
	Female (%)	10 (33.3)	23 (27.7)	–
Laboratory data				
	WBC (x10^3^/µL)	6.1 ± 1.9	6.3 ± 2.2	.784
	Hb (g/dL)	12.8 ± 1.7	12.9 ± 1.5	.752
	Plt (×10^4^/µL)	23.4 ± 6.5	23.9 ± 8.0	.948
	PT (%)	94.8 ± 21.7	94.2 ± 22.4	.971
	Alb (g/dL)	3.9 ± 0.4	4.0 ± 0.4	.539
	T-bil (mg/dL)	1.1 ± 0.9	1.9 ± 3.3	.507
	AST (IU/L)	50.3 ± 54.2	58.1 ± 47.4	.069
	ALT (IU/L)	61.9 ± 74.7	76.3 ± 76.0	.195
	ChE (IU/L)	245.7 ± 68.1	246.0 ± 58.0	.911
	CRP (mg/dL)	1.0 ± 1.5	0.8 ± 1.4	.883
	HbA1c (%)	6.1 ± 0.9	5.7 ± 0.9	.093
Liver function				
	ICGR15 (%)	9.8 ± 4.3	12.1 ± 9.1	.196
	HH15	0.562 ± 0.050	0.570 ± 0.068	.962
	LHL15	0.924 ± 0.032	0.932 ± 0.026	.374

*P*-values were determined using the Chi-square test or Mann–Whitney *U*-test. Values in bold indicate significant differences (*P* < .05).

ALT = alanine aminotransferase, AST = aspartate aminotransferase, ChE = cholinesterase, CRP = C-reactive protein, Hb = hemoglobin, HbA1c = hemoglobin A1c, HH15/LHL15, =hepatic uptake/liver to heart ratio at 15 min of technetium 99m diethylenetriaminepentaacetic acid galactosyl human serum albumin scintigraphy, ICGR15 = indocyanine green retention rate at 15 minutes, T-bil = total bilirubin, WBC = white blood cell count.

### 3.2. Primary diseases

The primary diseases were PHCC, hepatocellular carcinoma, intrahepatic cholangiocarcinoma, gallbladder carcinoma, colorectal cancer metastases as malignant tumors, and alveolar echinococcosis as a benign disease. PHCC was most common in both older group (n = 20, 66.8%) and younger group (n = 41, 49.4%), followed by hepatocellular carcinoma (13.3% and 20.5%, respectively; Supplemental Digital Content 1, Supplemental Digital Content, https://links.lww.com/MD/R737).

### 3.3. Surgical procedures

Among patients who underwent PVE, we performed right or left trisectionectomy, right or left hepatectomy, and exploratory laparotomy for patients with peritoneal dissemination. Right hepatectomy was most common in both older (n = 26, 86.7%) and younger groups (n = 60, 72.3%). There were no exploratory laparotomies or unresectable cases in the older group; however, there were 4 cases of insufficient RLV and 4 cases of exploratory laparotomy with peritoneal dissemination in the younger group (Supplemental Digital Content 1, Supplemental Digital Content, https://links.lww.com/MD/R737).

### 3.4. Embolized portal vein branches

We performed embolization of various portal vein branches; however, the right main portal branch (anterior and posterior branches) was embolized most commonly in both older (n = 27, 90.0%) and younger groups (n = 71, 85.6%; Supplemental Digital Content 1, Supplemental Digital Content, https://links.lww.com/MD/R737).

### 3.5. Period from PVE to surgery

**Figure 1.**
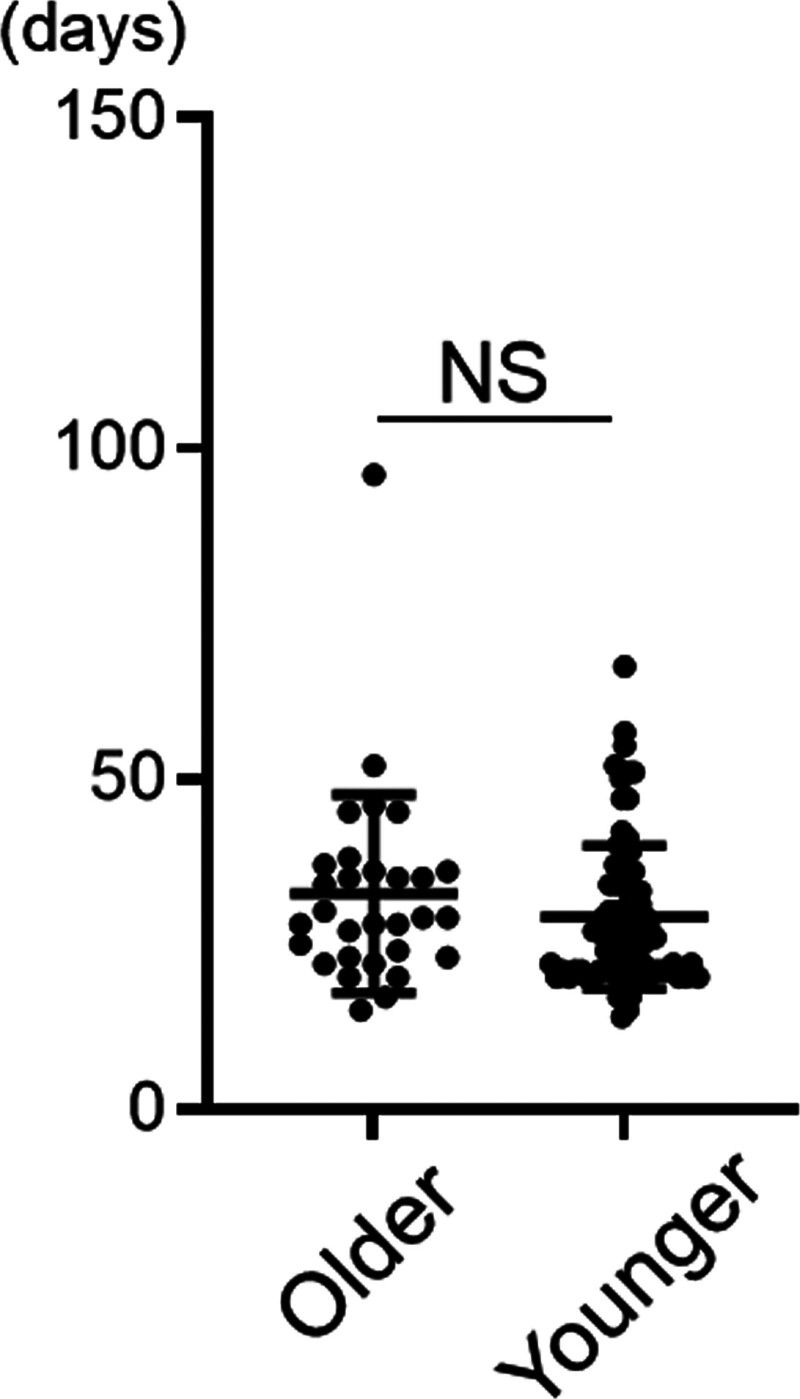
Period from PVE to surgery in older and younger patients. The median period from PVE to surgery was 29 (15–96) days in the older group and 26 (14–67) days in the younger group, with no significant difference between the groups. NS = no significant difference, PVE = portal vein embolization.

The median period from PVE to surgery was 29 (15–96) days in the older group and 26 (14–67) days in the younger group, with no significant difference between the groups (Fig. 1). Notably, all planned surgical procedures were performed in the older group.

### 3.6. Increase in RLV after PVE

**Figure 2.**
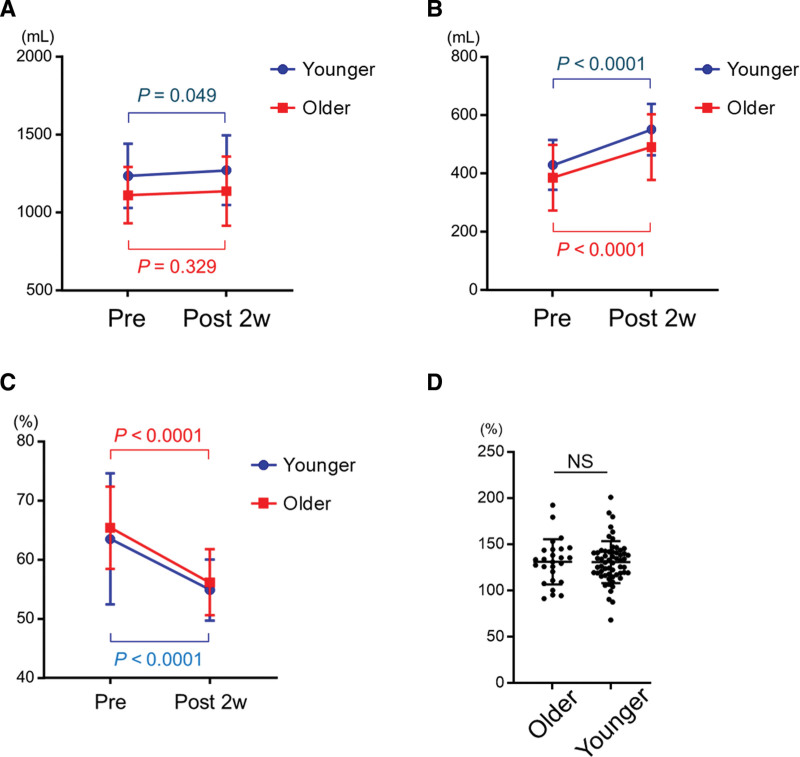
Increase in RLV after PVE in older and younger patients who underwent right hepatectomy. The total liver volume (TLV; range) increased slightly after PVE in the younger group (pre-/post-PVE TLV: 1220 (837–1855) mL/1256 (890–1887) mL; *P* = .049), but no significant change was observed in the older group (pre-/post-PVE TLV: 1090 (839–1539) mL/1100 (821–1824) mL; *P* = .329) (A). In comparison, the median (range) RLV increased 2 weeks after PVE in both groups (pre-/post-PVE RLV: 366 (234–683) mL/488 (311–796) mL, *P* < .0001 in the older group, and 419 (275–634) mL/532 (375–766) mL, *P* < .0001 in the younger group) (B). Although the median effective liver resection rate (ELRR) was ≥60% before PVE, the rate was <60% after PVE in both groups (pre-/post-PVE ELRR: 67.2% (48.2%–71.3%)/56.1% (44.1%–69.5%), *P* < .0001 in the older group and 64.5% (49.7%–75.2%)/54.5% (41.8%–63.4%), *P* < .0001 in the younger group) (C). The median increase in RLV 2 weeks after PVE was 131.9% (91.2%–192.3%) in the older group and 130.3% (68.1%–200.8%) in the younger group, indicating an obvious increase; however, no significant difference was observed between the 2 groups (D). The blue line indicates values for the younger group, and the red line indicates values for the older group. NS = no significant difference. PVE = portal vein embolization; RLV = remnant liver volume.

Because the RLV varies depending on the surgical procedure, we examined data for 86 patients (26 patients in the older group and 60 patients in the younger group) who underwent right hepatectomy, which was the most common surgical procedure in this study, to evaluate liver hypertrophy induced by PVE with the same surgical procedure. Although total liver volume (TLV) increased slightly in the younger group after PVE (pre-/post-PVE TLV: 1220 (837–1855) mL/1256 (890–1887) mL; *P* = .049), no significant change was observed in the older group (pre-/post-PVE TLV: 1090 (839–1539) mL/1100 (821–1824) mL; *P* = .329; Fig. 2A). In comparison, the median RLV increased significantly 2 weeks after PVE in both groups (pre-/post-PVE RLV: 366 (234–683) mL/488 (311–796) mL, *P* < .0001 in the older group and 419 (275–634) mL/532 (375–766) mL, *P* < .0001 in the younger group; Fig. 2B and Supplemental Digital Content 2, Supplemental Digital Content, https://links.lww.com/MD/R737. Although the median ELRR was ≥60% before PVE, the rate was <60% after PVE in both groups (pre-/post-PVE ELRR: 67.2% (48.2%–71.3%)/56.1% (44.1%–69.5%), *P* < .0001 in the older group, and 64.5% (49.7%–75.2%)/54.5% (41.8%–63.4%), *P* < .0001 in the younger group; Fig. 2C and Supplemental Digital Content 2, Supplemental Digital Content, https://links.lww.com/MD/R737). The median increase in RLV 2 weeks after PVE was 131.9% (91.2–192.3%) in the older group and 130.3% (68.1–200.8%) in the younger group, indicating an obvious increase, with no significant difference between the groups (Fig. 2D).

We also evaluated the association between pre-PVE FLR volume and the absolute change in FLR volume before and after PVE. This analysis demonstrated that, in both older and younger patients, a smaller pre-PVE FLR volume was inversely correlated with an absolute increase in FLR volume before and after PVE (*r* = −0.42, *P* = .021 in the older group and *r* = −0.30, *P* = .006 in the younger group; Fig. S1, Supplemental Digital Content, https://links.lww.com/MD/R736).

### 3.7. Complications

Regarding PVE-related complications, recanalization of the embolic portal branch was observed in 2 patients (6.7%) in the older group and 11 patients (13.3%) in the younger group, and arterial puncture occurred in 1 patient (3.3%) in the older group; this patient recovered without additional treatment. Regarding surgery-related complications, there were 6 cases (20.0%) in the older group and 11 cases (13.3%) in the younger group of postoperative grade B liver failure in accordance with the International Society for Study of Liver Surgery grade, with 1 case of Grade C in each group (Table 2).

**Table 2:** Complications related to PVE or liver surgery.

		Older	Younger	*P*-value
		n = 30	n = 83	
Related to PVE				
	Recanalization (n, %)	2 (6.7%)	11 (13.3%)	.263
	Arterial puncture (n, %)	1 (3.3%)	0 (0%)	.091
Related to surgery				
	postoperative liver failures (ISGLS)			
	Grade B (n, %)	6 (20.0%)	11 (13.3%)	.352
	Grade C (n, %)	1 (3.3%)	1 (1.2%)	.454

*P*-values were determined using the Chi-square test or Mann–Whitney *U*-test. Postoperative liver failure was graded in accordance with the International Society for Study of Liver Surgery (ISGLS) grade.

ISGLS = International Society for Study of Liver Surgery, PVE = portal vein embolization.

### 3.8. Univariate and multivariate analyses of parameters associated with the increase in RLV after PVE

Univariate and multivariate analyses showed that a high platelet count (odds ratio (OR): 10.89, 95% Confidence intervals (CI): 1.37–235.06; *P* = .047) and high PV flow measured before PVE (OR: 16.62, 95% CI: 1.95–392.14; *P* = .025) were independent factors affecting the increase in RLV after PVE in the older group (Table 3). In contrast, in the younger group, high serum albumin (OR: 2.95, 95% CI: 1.21–7.44; *P* = .017) and high cholinesterase (OR: 2.85, 95% CI: 1.17–7.20, *P* = .021) were favorable factors affecting the increase in RLV after PVE in the univariate analyses, although there was no statistical significance in the multivariate analysis (Supplemental Digital Content 3, Supplemental Digital Content, https://links.lww.com/MD/R737). In the entire cohort, high WBC count (OR: 3.43, 95% CI: 1.18–11.58; *P* = .023) and high cholinesterase (OR: 2.63, 95% CI: 1.21–5.88, *P* = .015) were identified as independent factors in both the univariate and multivariate analyses, whereas age was not identified as an independent factor affecting the increase in RLV after PVE (Supplemental Digital Content 4, Supplemental Digital Content, https://links.lww.com/MD/R737).

**Table 3:** Univariate and multivariate analyses of the increase in liver volume in older patients who underwent PVE.

Variables	Increase of RLV			
	Univariate analysis		Multivariate analysis	
	OR (95% CI)	*P*-value	OR (95% CI)	*P*-value
Age > 74 yr		-		
Gender: male	0.23 (0.04–1.11)	0.068		
WBC > 7500	6.00 (0.75–126.90)	0.094		
Neu/Lym > Ave	0.67 (0.13–3.07)	0.604		
Hb > 13.1	0.78 (0.18–3.303)	0.732		
Plt > 23.9*	**5.40 (1.19–28.98**)	**0.028**	**10.89 (1.37–235.06**)	**0.047**
PT > 80	5.91 (0.79–122.45)	0.086		
Alb > 4.0	0.56 (0.12–2.38)	0.430		
T-bil > 1.0	0.45 (0.08–2.22)	0.334		
AST > 38	1.67 (0.39–7.43)	0.490		
ALT > 44	1.65 (0.37–7.69)	0.510		
ChE > 250	1.67 (0.39–7.43)	0.490		
CRP > 0.3	1.33 (0.31–5.8)	0.695		
HbA1c > 5.6	0.90 (0.19–4.26)	0.893		
ICGR15 > 10.0	1.25 (0.28–5.54)	0.765		
HH15 > Ave	1.08 (0.24–4.95)	0.919		
LHL15 > Ave	0.80 (0.18–3.50)	0.765		
**PV flow (pre PVE) > Ave**	**10.50 (1.74–94.31**)	**0.009**	**16.62 (1.95–392.14**)	**0.025**
PV flow (3days after PVE) > Ave	4.81 (0.95–28.85)	0.058		
Rate of increase of PV flow (pre-post) > Ave	0.43 (0.07–2.27)	0.323		
Pressure of PV at pre PVE	0.45 (0.08–2.25)	0.335		
Pressure of PV at post PVE	0.50 (0.08–2.63)	0.417		
Rate of increase of PV pressure (pre-post) > Ave	0.33 (0.06–1.70)	0.187		

Values in bold indicate significant differences (*P* < .05). The optimal cutoff value for the platelet count (asterisk) was defined as the lowest value for (1 − sensitivity)2 + (1 − specificity)^2^, which corresponded to values with sensitivity and specificity closest to the (0, 1) point on the receiver operating characteristic curve. The optimal cutoff values for factors other than the platelet count were defined as the normal values in our hospital.

Alb = serum albumin, ALT = alanine aminotransferase, AST = aspartate aminotransferase, Ave = average, ChE = cholinesterase, CI = confidence interval, CRP = C-reactive protein, Hb = hemoglobin, HbA1c = hemoglobin A1c, HH15/LHL15 = hepatic uptake/liver to heart ratio at 15 min of technetium 99m diethylenetriaminepentaacetic acid galactosyl human serum albumin scintigraphy, ICGR15 = indocyanine green retention rate at 15 minutes, Lym = lymphocytes, Neu = neutrophils, OR = odds ratio, Plt = platelet count, PT = prothrombin time, PV = portal vein, PVE = portal vein embolization, T-bil = total bilirubin, WBC = white blood cell count.

## 4. Discussion

Our results showed that even in older patients aged ≥75 years, the increase in RLV after PVE was comparable to that in young people. Additionally, all planned surgical procedures were performed in the older group, and it was possible to safely perform preoperative PVE without severe complications related to PVE. These findings suggest that preoperative PVE may be feasible and safe in selected older patients.

Complete resection of hepatobiliary malignant tumors is the best method to achieve long-term survival.^[19]^ However, post-hepatectomy liver failure remains a significant cause of morbidity and mortality after major liver resection.^[4]^ Accordingly, the 2006 expert consensus statement recommends a minimum safe limit of FLR 20% for liver resection with a normal liver.^[20]^ However, the guidelines are less clear for patients with chronic liver disease or older people, where the safe extent of resection depends on both the nature and severity of the underlying disease.^[21,22]^ Some reports showed that the safe FLR limit for patients with chronic liver diseases, such as mild steatosis, cholestasis, and early cirrhosis, ranges from 30% to 40%.^[23-25]^ Therefore, in patients with borderline FLR, volume optimization strategies, such as PVE, must be considered before hepatectomy. PVE is the most widely used volume optimization strategy and is indicated for patients requiring extensive hepatectomy with borderline or insufficient FLR volume.^[26]^ Preoperative PVE is technically feasible in over 90% of patients and carries a low risk of complications.^[13]^ PVE is available worldwide as an effective technique to optimize FLR before major hepatectomy for any indication.^[27,28]^ Guidelines state acceptable complication rates of <25% for minor complications and <5% for major complications.^[29,30]^ The complications rate in our cohort met these criteria.

The mechanism of liver regeneration after PVE is complex, and the exact trigger remains elusive. Previous reports have suggested that the combination of periportal inflammation in the embolized liver and the diversion of PV blood to the FLR are important stimuli for regeneration.^[31]^ We previously reported that preserved liver function and increased PV flow were important for hepatic hypertrophy after PVE.^[18]^ Additionally, the univariate and multivariate analyses in the current study indicated that greater inflow through the portal vein to the residual liver was associated with greater increases in FLR volume in older people. The loss of liver regenerative capacity with aging creates serious problems in postoperative recovery in older patients, and various factors are involved. Takubo et al reported progressive telomere shortening with aging in the normal human liver,^[32]^ and Aini et al revealed accelerated telomere shortening relative to graft age after transplantation, probably reflecting high cell turnover and oxidative stress.^[33]^ Schmucker et al reviewed the effect of aging on the hepatocellular response to growth factors.^[34]^ The authors observed an 80% age-related decline in the amount of radiolabeled epidermal growth factor associated with rat hepatocyte nuclei. In our study, favorable factors for increased FLR volume were inconsistent between older and younger groups. However, the increase in FLR volume in older patients was similar to that of younger patients. Specifically, the higher FLR volume in older patients compared with that in younger patients may have resulted from the higher platelet count and higher PV inflow volume to the residual liver. Moreover, PV inflow volume to the residual liver might be a surrogate marker for a potential increase in FLR volume after PVE in older patients. In addition, a smaller pre-PVE FLR volume was associated with a greater absolute increase in FLR volume in both older and younger patients, suggesting a compensatory regenerative response (Fig. S1, Supplemental Digital Content, https://links.lww.com/MD/R736).

The impact of aging on PVE in hepatectomy for PHCC has been reported.^[12]^ The authors showed that aging suppressed hepatic reserve in the FLR after PVE using indocyanine green clearance and concluded that preoperative PVE should be performed with attention to the influence of aging on hepatic reserve in older patients with PHCC. However, the authors also reported that post-hepatectomy liver failure grade A was observed in only 1 (4%) patient, and no mortality was observed after 30 or 90 days. These results suggest that, although aging suppressed hepatic reserve, hepatectomy can be performed safely using preoperative PVE in older patients with PHCC, which is consistent with our results. Regarding hepatocellular carcinoma, we previously reported that hepatectomy can be performed safely, even in patients >80 years of age, if the tumor is small and adequate liver function reserve is present.^[35]^ These patients can expect the same favorable prognoses as those of young individuals.^[35]^ Preoperative PVE is crucial for safe hepatectomy and to preserve remnant liver reserve, which is important to achieve a good prognosis in older patients.

Regarding the period from performing PVE to achieving sufficient hepatic reserve, hepatocyte regeneration occurs at rates ranging from 21 cm^3^/d at 2 weeks to 11 cm^3^/d at 4 weeks.^[36]^ We previously demonstrated that FLV increase after PVE began 1 week post-PVE and plateaued after 3 weeks.^[37]^ The optimal timing of surgery is considered to be up to 4 weeks after PVE because major hepatectomy is usually planned 4 to 6 weeks after PVE; no significant hypertrophy is seen after 6 weeks.^[38]^ In this study, the median period from PVE to surgery was 29 days in older patients, comparable to 26 days in younger patients, and all planned hepatectomies were performed in the older group.

There are limitations in this study. First, this was a retrospective single-center study, and the number of older patients was low compared with the number of younger patients. Second, older patients who underwent PVE and major hepatectomy tended to have better performance status compared with younger patients due to possible more carefully selection, despite the lack of a significant difference in the patients’ characteristics. This finding may have resulted from physicians excluding older patients who were not in good general health. Additional studies of larger cohorts and prospective studies are required to reveal the mechanism underlying the influence of PVE on major hepatectomy in older people.

## 5. Conclusions

In this cohort study, even in patients aged ≥75 years, the increase in RLV after PVE was comparable to that in younger patients. Moreover, all planned surgical procedures for older patients were performed without complications related to PV embolization. PVE may be useful and can be performed safely prior to major hepatectomy in appropriately selected older patients.

## Acknowledgments

We thank Jane Charbonneau, DVM, from Edanz (https://jp.edanz.com/ac) for editing a draft of this manuscript.

## Author contributions

**Conceptualization:** Yuzuru Sakamoto, Shingo Shimada, Toshiya Kamiyama.

**Data curation:** Yuzuru Sakamoto, Shingo Shimada, Yoh Asahi, Akihisa Nagatsu, Tatsuya Orimo, Tatsuhiko Kakisaka, Hirofumi Kamachi.

**Formal analysis:** Yuzuru Sakamoto, Shingo Shimada.

**Investigation:** Yuzuru Sakamoto.

**Methodology:** Yuzuru Sakamoto, Shingo Shimada, Toshiya Kamiyama.

**Supervision:** Akinobu Taketomi.

**Writing – original draft:** Yuzuru Sakamoto.

**Writing – review & editing:** Shingo Shimada, Toshiya Kamiyama, Tatsuhiko Kakisaka, Daisuke Abo, Akinobu Taketomi.

## Supplementary Material




