# Supplementary material for: Usefulness and safety of preoperative portal vein embolization in older adults: A STROBE-compliant observational study
Source: Medicine (Baltimore). 2026 Apr 24;105(17):e48457. doi: 10.1097/MD.0000000000048457 (PMC13124379; doi:10.1097/MD.0000000000048457)

Visual Abstract :

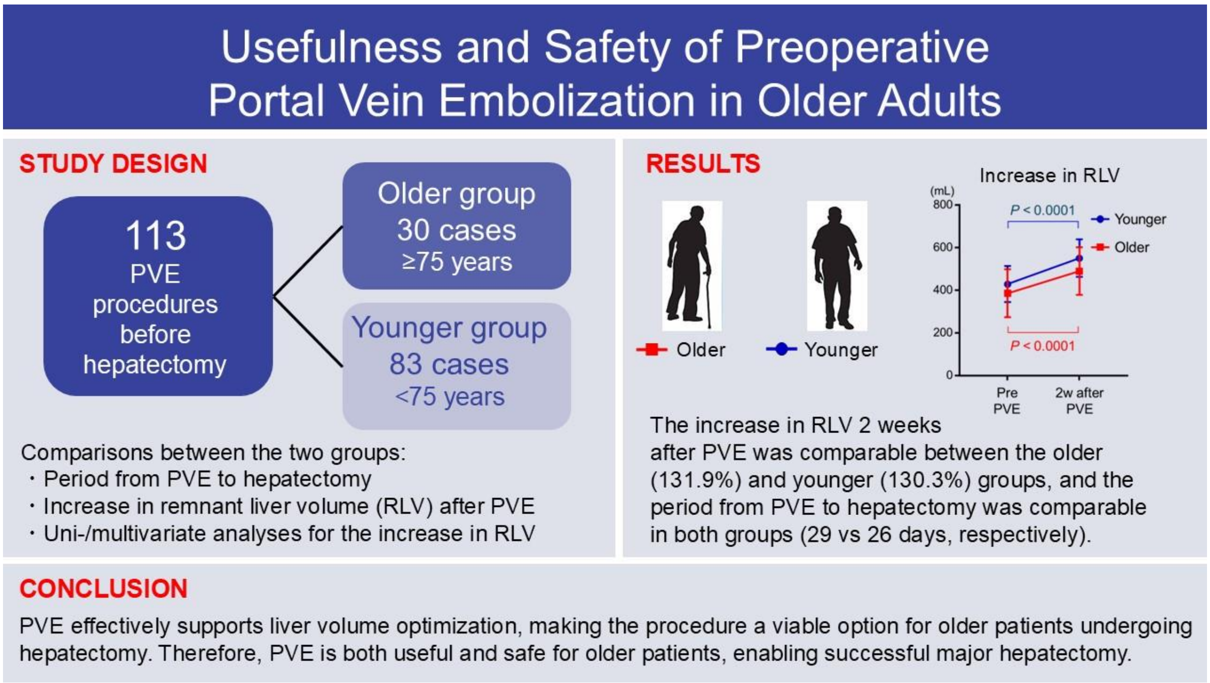

Supplementary Figure 1. Correlations between Pre-PVE FLR volume and Δ FLR volume in all, older and younger patients.

A smaller pre-PVE FLR volume was inversely correlated with an absolute increase in FLR volume before and after PVE (r = -0.42, P = 0.021 in the older group, r = -0.30, P = 0.006 in the younger group, and r = -0.32, P < 0.001 in all patients).

The Δ FLR volume was calculated by subtracting the Pre-PVE FLR volume from the FLR volume measured 2 weeks after PVE. P values and correlation coefficients (r) were calculated with Spearman's correlation test. FLR, future liver remnant; PVE, portal vein embolization.

Supplementary Figure1

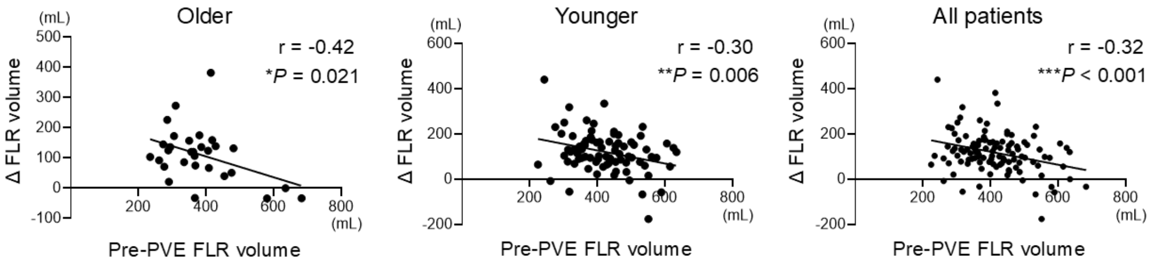

Supplement: Supplementary file 2 [file medi-105-e48457-s002.pdf]
